# Advances in Instrumental Analysis of Brominated Flame Retardants: Current Status and Future Perspectives

**DOI:** 10.1155/2014/651834

**Published:** 2014-10-28

**Authors:** Mohamed Abou-Elwafa Abdallah

**Affiliations:** ^1^Division of Environmental Health and Risk Management, School of Geography, Earth and Environmental Sciences, University of Birmingham, Birmingham B15 2TT, UK; ^2^Department of Analytical Chemistry, Faculty of Pharmacy, Assiut University, Assiut 71526, Egypt

## Abstract

This review aims to highlight the recent advances and methodological improvements in instrumental techniques applied for the analysis of different brominated flame retardants (BFRs). The literature search strategy was based on the recent analytical reviews published on BFRs. The main selection criteria involved the successful development and application of analytical methods for determination of the target compounds in various environmental matrices. Different factors affecting chromatographic separation and mass spectrometric detection of brominated analytes were evaluated and discussed. Techniques using advanced instrumentation to achieve outstanding results in quantification of different BFRs and their metabolites/degradation products were highlighted. Finally, research gaps in the field of BFR analysis were identified and recommendations for future research were proposed.

## 1. Introduction

Flame retardants are a diverse group of chemicals added to a wide range of consumer products, including plastics, polymers, textiles, building materials, and electric and electronic equipment, to prevent or delay the propagation of fire. Currently, there are 4 major groups of flame retardants on the market: inorganic, halogenated organic, organophosphorus, and nitrogen based compounds. Brominated flame retardants (BFRs; a subgroup of the halogenated organic class) are currently the largest market group of flame retardants due to their low cost and high performance efficiency [1]. In 2006, the total consumption of flame retardants in Europe was 465000 t, of which 10% were BFRs [2]. There are ~75 different commercial BFRs, each with specific properties depending on the nature of the material they are protecting. Some BFRs are reacted (i.e., chemically-bonded) into the final polymer while most of them are used as additives to the polymer matrix. Available figures show the most widely used BFRs are tetrabromobisphenol A (TBBP-A) with a global demand of 170,000 tonnes in 2004, alongside decabromodiphenyl ether (Deca-BDE), hexabromocyclododecane (HBCD), pentabromodiphenyl ether (Penta-BDE), and octabromodiphenyl ether (Octa-BDE), for which worldwide market demands in 2001 were 56,100, 16,700, 7,500, and 3,790 tonnes, respectively [3]. Since polybrominated diphenyl ethers (PBDEs) and HBCD (and ~20% of the production of TBBP-A) are blended physically rather than bonded chemically to polymeric materials, they migrate into the environment where their persistence and bioaccumulative characters lead to contamination of humans [4]. This is of concern owing to the potential health risks associated with human exposure to these compounds including endocrine disruption, neurodevelopmental, and behavioural disorders, hepatic abnormality, and possibly cancer [5, 6]. The few data available from human epidemiological studies imply effects on male reproductive hormones [7], semen quality [8], thyroid hormone homeostasis [9], and cryptorchidism [10], as well as lower birth weight and length [11]. Such evidence has contributed to complete EU bans for Penta and OctaBDE, and restrictions on the use of DecaBDE in addition to other restrictions within severaljurisdictions on the manufacture and new use of the three commercial PBDE formulations across the world [4]. Moreover, HBCD and PBDEs associated with Penta and OctaBDE have been listed under the UNEP Stockholm Convention on POPs, while DecaBDE is currently under consideration for listing under Annexes A, B, and/or C to the convention [12]. Despite such restrictions, human exposure to BFRs is likely to continue for the foreseeable future, given their persistence and ubiquity of flame-retarded products in the environment [13]. Furthermore, the restrictions on the production and usage of HBCD and PBDEs have paved the way for development and application of “novel” BFRs as replacements for the banned formulations. Important representatives of this NBFR group are decabromodiphenyl ethane (DBDPE), 1,2-bis(2,4,6-tribromophenoxy)ethane (BTBPE), 2-ethylhexyl-2,3,4,5-tetrabromobenzoate (TBB), and bis(2-ethylhexyl)-3,4,5,6-tetrabromo-phthalate (TBPH) (Table 1). While more information, especially regarding their toxicological profile, is required to define the fate and transport characteristics of NBFRs, the current state-of-knowledge on the production, usage, environmental occurrence, persistent (P), bioaccumulative (B), and toxic (T) characteristics of various NBFRs was recently reviewed [14]. Against the continuously increasing scientific interest in the environmental fate, behavior, and human health implications of the currently ubiquitous BFRs, one of the major trends in analytical chemistry is efficient determination of the trace levels of various BFRs in complex matrices [15]. Different aspects related to production, usage, environmental occurrence, toxicity, and human exposure to different BFRs have been recently reviewed [14, 16–20]. Therefore, the aims of this work are (a) to provide a critical review of the recent analytical techniques applied for the analysis of various classes of BFRs in different environmental and biological matrices and (b) to discuss the current challenges in the field of BFR analysis and provide recommendations for future research in this field.

## 2. Sample Preparation for BFR Analysis

Understanding the physicochemical properties of pollutants is pivotal to study their fate and behaviour in both indoor and outdoor environments. To this end, BFRs display a wide range of physicochemical properties depending on their molecular structure and weight (Table 1). The large variety in molecular weight, polarity, vapour pressure, and log⁡ K_ow_ displayed by different classes of BFRs is associated with varying degrees of environmental mobility, long-range transport, persistence, bioaccumulation, and toxicity [21]. Furthermore, the diversity in physicochemical parameters displayed by BFRs represents a continuous challenge for analytical chemists aiming to develop multiresidue methods for their analysis. Therefore, several methods were reported for exhaustive extraction and clean-up of different BFRs from both biotic and abiotic environmental matrices. Advances in sample preparation techniques for analysis of BFR in environmental matrices have been recently reviewed [22]. Hence, sample preparation techniques are not the focus of the current paper and will only be briefly summarized in Tables 2 and 3.

## 3. Instrumental Analysis of BFRs

Due to their diverse nature, wide range of physicochemical properties, large number of congeners and relatively low concentrations in various matrices, chromatographic separation hyphenated with mass spectrometric detection techniques are generally the method of choice for analysis of different BFRs in biotic and abiotic matrices.

### 3.1. GC/MS Analysis

GC/MS is the most commonly used technique for analysis of BFRs [54]. Thermal degradation and isomeric interconversion are the main challenges facing analytical chemists with the GC/MS analysis of BFRs. Therefore, several parameters of the GC/MS system need to be carefully optimised according to the properties of target analytes. These include injection technique, stationary phase, column dimensions, and mass spectrometric parameters.

#### 3.1.1. Sample Injection

Because of their relatively low levels in most matrices, the most common injection techniques applied for BFR analysis are splitless injection, on-column injection, and programmed temperature vaporisation (PTV) [55]. In addition to its low cost and availability as a standard feature for most GC/MS instruments, splitless injection is favoured by several analysts due to the expected trace levels of BFRs in most environmental samples. However, thermal degradation and mass discrimination of higher molecular weight compounds are main drawbacks of this technique [56]. Therefore, injector temperature and splitless time need to be optimised for maximum sensitivity. For instance, highest possible temperature and long splitless time (325°C for 4 min) resulted in an increased response factor of BDE-209 [57]. An alternative way to minimize thermal degradation in the injector/liner section of the instrument is direct on-column injection. In this technique, the injected sample is delivered directly to the entrance of the capillary column resulting in higher precision and less variability of the results [58]. However, extensive sample clean-up is required to prevent matrix-related interfering substances and macromolecular residues from reaching the column which may cause peak tailing, high noise levels, retention time shifts, and eventually shorten the column lifetime [57]. Recently, PTV injection emerged as the method of choice for multiresidue analysis of different classes of BFRs and NBFRs in the same sample. PTV can provide several advantages including minimal degradation of thermolabile contaminants, reduced thermal discrimination of high molecular weight compounds, large injection volumes, and improved response factor of higher molecular weight PBDEs [34, 59, 60].

#### 3.1.2. Stationary Phase and GC Column Dimensions

The elution order of 126 PBDE congeners was determined and compared on 7 different GC column stationary phases [61]. The most suitable stationary phase for efficient separation of PBDE congeners was DB-XLB (J&W Scientific) followed closely by DB-1 (J&W Scientific) column. However, the latter is usually preferred for routine PBDE analysis due to reduced degradation of higher brominated congeners. For NBFRs, low polarity stationary phases were generally used for their separation. The most commonly reported stationary phase for analysis of NBFRs composed of 5% phenyl; 95% dimethyl polysiloxane (e.g., DB5-MS from J&W), in most cases with a thin film thickness (0.1 0.25 *μ*m) [62]. This combination is particularly favoured due to short on-column residence time and reduced retention which is beneficial for high M.Wt compounds (e.g., DBDPE, BTBPE, and TBBPA-DBPE) or for NBFRs that are prone to on-column thermal decomposition or isomer interconversion [62]. However, single dimension GC cannot separate all PBDE congeners even with the most efficient stationary phase (22 coelutions were observed on a DB-XLB phase [61]). Therefore, Korytár et al. evaluated 6 column combinations for 2 dimensional GC × GC separation of PBDEs. Results revealed that a DB-1 × 007-65HT (Quadrex) combination was the most suitable combination because of (a) the highest number of PBDE congeners separated, (b) less decomposition of higher brominated congeners, and (c) most suitable maximum operating temperature [63].

Generally, short columns (10–15 m) are currently used for routine analysis of major PBDEs and NBFRs. while these columns provide the advantage of minimal thermal degradation and isomerisation of high M.Wt BFRs (e.g., BDE-209 and DBDPE), coelution of TBB with BDE 99 has been described using this type of column [64]. Longer GC columns (25–60 m) were applied to achieve better analyte separation, especially when NBFRs and PBDEs were simultaneously analysed or for confirmation purposes [65, 66]. Vetter and Rosenfelder reported on the retention data of 122 environmentally-relevant polybrominated compounds including PBDEs, HBCDs, and NBFRs using a 30 m HP-5MS column. Potential coelutions were reported and discussed including that of Allyl 2,4,6-tribromophenyl ether (ATE) with BDE-10 [67]. Phenolic BFRs usually require derivatisation prior to injection onto the relatively nonpolar columns used for BFR analysis. Phenolic NBFRs including TBBP-A, 2,4-dibromophenol (2,4-DBP), 2,4,6-tribromophenol (TBP), and pentabromophenol (PBP) were successfully separated on a 25 m CPSil-8 column following derivatisation with acetic anhydride [68]. A 30 m HP-1 column was used for the analysis of 2,4-DBP, 2,4,6-TBP, and PBP following their silylation with bis-(trimethylsilyl)-trifluoroacetamide (BSTFA) [69]. A method for the simultaneous determination of underivatised phenolic BFRs as well as their byproducts, formulation intermediates, and decomposition products was reported using a 60 m CPSil-8-CB column [70]. GC/MS methods could not be used for diastereomer- or enantiomer-specific analysis of HBCDs due to isomeric interconversion at temperatures >160°C [71].

#### 3.1.3. Mass Spectrometric Detection

**Figure 1:**
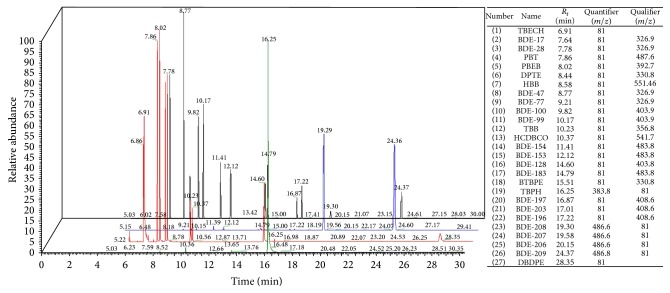
3D-stacked GC-ECNI/MS chromatograms of 0.5 ng/*μ*L standard mixtures of various PBDEs and NBFRs.

Both high resolution (HR) and low resolution (LR) single quadrupole mass spectrometers have been widely applied for detection and quantification of PBDEs and their methoxylated derivatives [72, 73]. The LR/MS instruments could be operated in either electron ionization (EI) or negative chemical ionization (NCI) mode. In EI/MS, the major ions reported for PBDE analysis were [M]^+^ and [M − 2Br]^+^ [74]. While this can provide more selectivity for identification and structural confirmation of target PBDEs, LR-EI/MS is not commonly used for analysis of higher PBDEs (more than 6 Br atoms) due to reduced sensitivity. For instance, GC-EI/MS operated in SIM mode was successfully applied for analysis of PBDEs in human hair samples with LOQ as low as 0.3–0.6 ng/g for tri- to hepta-BDEs and 3 ng/g for BDE-209 [75]. Therefore, NCI, also known as ECNI (electron capture negative ionisation), has been more widely used for determination of high M.Wt. PBDEs. Most PBDEs (except for BDE-209) do not produce abundant stable molecular or fragment ions in the ECNI source; hence, only bromide ions (*m*/*z* 79 and 81) can be monitored. This reduced selectivity of the ECNI source precludes the use of ^13^C-labelled PBDEs as internal standards, except for BDE-209 which produces a stable [C_6_Br_5_O]^−^ fragment (*m*/*z* 486.7) allowing for the use of ^13^C-BDE-209 as internal standard [58]. Nevertheless, the high sensitivity of GC-ECNI/MS rendered it the most commonly used method for analysis of major PBDEs in addition to other NBFRs (Figure 1) in various biotic and abiotic matrices [18, 62]. Furthermore, selectivity of GC-ECNI/MS can be improved via optimisation of the electron energy, emission current, source temperature, and system pressure to increase the relative abundances of larger molecular fragments [M − *x*H − *y*Br]^−^ which enables the monitoring of each PBDE homologue group rather than the non-specific bromide ions [76]. Monitoring high mass fragments of PBDEs under optimised ion source conditions was successfully applied for analysis of PBDEs in snow and human serum samples at concentration levels <0.01 pg/mL [77].

While bromide ions (*m*/*z* 79, 81) were usually monitored for most NBFRs in GC-ECNI/MS [78], other ions were occasionally reported for specific compounds. For example, TBPH was analyzed via monitoring molecular fragments at *m*/*z* 463, 461 [79] and *m*/*z* 463, 515 [64]. In addition, the coelution of TBB with BDE-99 rendered it necessary to use fragment ions (*m*/*z* 357, 471) for its monitoring to improve method selectivity [64]. Although not available to most laboratories, gas chromatography–high resolution mass spectrometry in EI mode (GC-HR-EI-MS) was applied successfully for detection and quantification of a wide range of NBFRs including ATE, TBCO, TBB, BATE, PBEB, DPTE, HBB, HCDBCO, DP, TBECH, BTBPE, BEHTBP, OBIND, and DBDPE [80].

Recently, further advanced MS techniques were applied for multiresidue analysis of BFRs. GC- time of flight (TOF)-MS was applied for analysis of PBDEs, along with PCBs, in soil samples with LODs of 0.1–0.6 ng/g dry weight [81]. A method based on GC-MS/MS was described for analysis of PBDEs, along with PCBs and organochlorine pesticides (OCs), in human breast tissues with LODs as low as 0.5 ng/g (50 ng/g for BDE-209). Analyses were performed in both EI-selected reaction monitoring (SRM) mode and NCI-selected ion recording (SIR) mode where the acquisition of—at least—2 SRM transitions (in EI) or ions (in NCI) per analyte allowed positive findings to be confirmed by accomplishment of ion ratios between the quantification and the confirmation transitions or ions [82]. Another GC coupled to ion trap tandem mass spectrometry (GC-IT-MS/MS) method was successfully applied for monitoring a wide range of PBDEs and NBFRs, together with Dechlorane plus, in the blubber of harbor porpoises. The method achieved low LODs (<1 ng/g lipid weight) and high precision (RSD < 15%) for all target analytes [50]. Another interesting approach was the use of comprehensive two-dimensional gas chromatography coupled to atmospheric pressure chemical ionization-high resolution time-of-flight-mass spectrometry (GC × GC-APCI-TOF/HRMS) for analysis of a wide range of BFRs and plasticizers with absolute LODs in the range 0.5–25 pg. The method took the advantage of using a soft ionization technique that provides mainly molecular ions, in addition to the accuracy of HRMS for identification of a wide range of compounds. The application of direct probing provided a very easy and inexpensive method for the identification of flame retardants without any sample preparation. This technique seems extremely useful for the screening of solid materials such as electrical devices, electronics, and other waste [83].

### 3.2. LC/MS Analysis

The inherent problems encountered with GC/MS analysis due to the high temperatures applied resulted in several difficulties in the analysis of some BFRs. Particularly, HBCDs where isomeric interconversion takes place at temperatures >160°C rendering isomeric separation impossible on GC columns [19]. Another problem encountered with GC/MS analysis of BFRs is thermal decomposition of high molecular weight compounds (e.g., BDE-209, DBDPE, and TBBPA-DBPE) [84]. While the use of ^13^C-BDE-209 as surrogate standard can—to some extent—account for the inevitable on-column degradation of BDE-209 during GC-NCI/MS analysis, similar approaches could not be achieved for DBDPE and TBBPA-DBPE due to the lack of stable molecular fragments other than *m*/*z* = 81 in the NCI source [84, 85]. Furthermore, the increased interest in relatively polar compounds such as TBBP-A and hydroxylated PBDE metabolites meant that a derivatisation step is required prior to their GC/MS analysis which may result in significant analyte loss and reduced recoveries [16, 86]. Therefore, LC/MS analysis emerged as an alternative technique to avoid the problems encountered during the analysis of thermolabile and relatively polar compounds by GC/MS.

#### 3.2.1. HBCD

HBCD is produced via bromination of cyclododeca-1,5,9-triene (CDT) resulting in the creation of six stereo centers at positions 1, 2, 5, 6, 9, and 10 of the formed product. This can give rise to a total of 16 possible optical isomers, 6 pairs of enantiomers, and 4* meso* forms. To date, only 3 diastereomers—named *α*-, *β*-, and *γ*-HBCD—were detected in the technical formulations and environmental samples with minor contributions (up to 0.5%) of two* meso* forms named *δ*- and *ε*-HBCDs [87].

(*1) Diastereomer-Specific Analysis*

**Figure 2:**
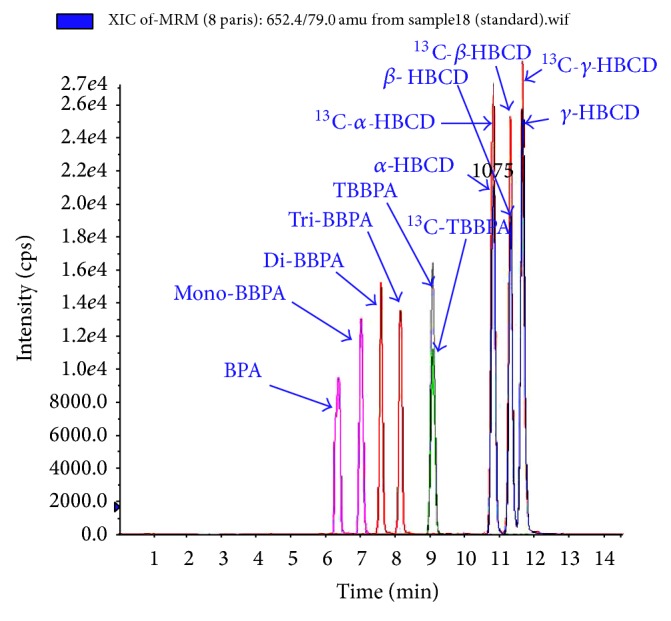
LC-ESI-MS/MS chromatogram of 0.5 ng/*μ*L standard mixtures of BPA, mono-BBPA, di-BBPA, tri-BBPA, TBBPA, and *α*-, *β*-, and *γ*-HBCDs.

*Stationary Phase.* Tomy et al. [88] reported on baseline separation of HBCD diastereomers on a C_18_-reversed phase column. While a 5 *μ*m particle size column (*Vydac 218MS, Mandel Scientific, Guelph, ON, Canada*) packing is sufficient for baseline separation of *α*-, *β*-, and *γ*-HBCDs, better resolution with sharper peaks (Figure 2) was reported using 3 *μ*m particle size (*Pursuit XRS3, Agilent, CA, USA*) [89]. Shorter retention times (<8 min) with narrower peaks could be achieved with C_18_  UPLC columns with 1.8 *μ*m particles (*Acquity HSS T3, Waters, MA, USA*) [90]. Moreover, separation of *α*-, *β*-, *γ*-, *δ*-, and *ε*-HBCDs was achieved on a 1.7 *μ*m UPLC column (*Acquity UPLC BEH, Waters, MA, USA*) [91].

*Mobile Phase.* Several mobile phase gradients using different combinations of methanol/acetonitrile/water were reported for separation of HBCD diastereomers [19]. While the MS response of an ESI source for 200 pg/*μ*L solution of HBCDs in methanol was reported to be slightly higher than that in acetonitrile [92], addition of acetonitrile to the mobile phase (up to 20%) resulted in improved resolution of the 3 main HBCD diastereomers (mainly between *β*- and *γ*-isomers) which is recommended when the 2 minor* meso* forms (*δ*- and *ε*-HBCD) are to be monitored [91]. Different mobile phase modifiers (e.g., ammonium acetate [93], ammonium chloride [94], and acetic acid [95]) were reported to produce sharper peaks and improve separation efficiency of HBCDs.

*(2) Enantiomer-Specific Separation*

**Figure 3:**
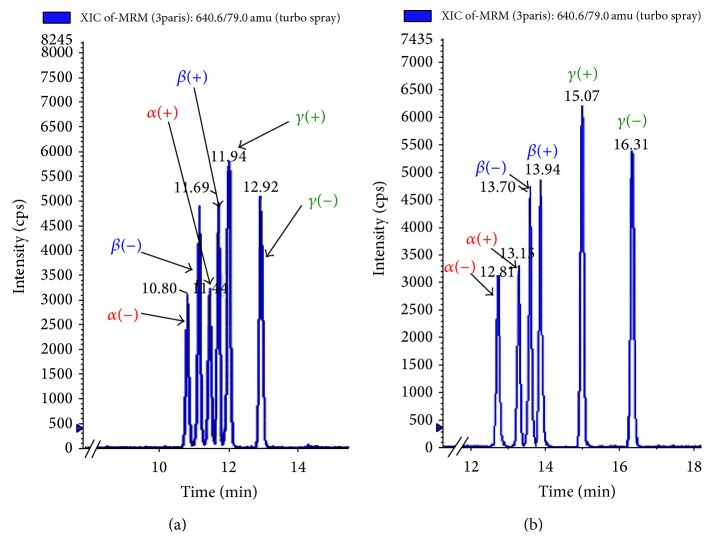
LC-MS/MS chromatograms showing chiral separation of 50 pg/*μ*L *α*-, *β*-, *γ*-HBCDs using (a) Nucleodex *β*-PM chiral LC column and (b) Pursuit XRS3 C18 column followed by Nucleodex *β*-PM chiral LC column.

*Stationary Phase.* Only one chiral stationary phase was reported in literature for efficient separation of HBCD enantiomers. Baseline resolution of the 6 enantiomers from an *α*-, *β*-, and *γ*-HBCD mixture was achieved on *β*-permethylated cyclodextrin bonded (*NUCLEODEX, Macherey-Nagel, GmbH, Düren, Germany*) chiral LC column (4 × 200 mm, 5 *μ*m) [96]. It was observed that (−)-*α*- and (−)-*β*-HBCD eluted before their corresponding (+)-*α*- and (+)-*β*-HBCD enantiomers (Figure 3). These were followed by the *γ*-enantiomers with (+)-*γ*- eluting ahead of (−)-*γ*-HBCD [97]. While the chiral column is sufficient for baseline separation of HBCD enantiomers, Yu et al. [98] connected a C_18_ achiral column to the *β*-permethylated chiral stationary phase in order to separate the HBCD diastereomers prior to enantiomeric resolution. This provided clear distinction between the respective enantiomers of each HBCD diastereomer in the resulting chromatograms (Figure 3).

*Mobile Phase.* A combination of methanol/acetonitrile/water in the mobile phase is mandatory for separation of HBCD enantiomers [99]. Interestingly, Marvin et al. [100] found that both mobile phase composition and column bleed could affect the MS response for different HBCD enantiomers. Dodder et al. [101] observed that the MS response changed between the elution of two enantiomers due to the extracted matrix component. In order to avoid such effects on the estimated enantiomeric fractions (EF), Marvin et al. [85] introduced a mathematical formula for calculation of corrected EF values (see (1). This correction is based on the use of isotopic-labelled standards (e.g., d_18_-HBCDs) since the labelled enantiomeric analogs behave identically to their native counterparts in the MS source [100]:

(1) $$\begin{array}{c} {\text{EF}}_{\text{corrected}}=\frac{\left[\left(A^{+}/A_{\text{labeled}}^{+}\right)\times \left(\text{pg}A_{\text{labeled}}^{+}\right)\right]}{\left[\left(A^{+}/A_{\text{labeled}}^{+}\right)\times \left(\text{pg}A_{\text{labeled}}^{+}\right)\right]+\left[\left(A^{-}/A_{\text{labeled}}^{-}\right)\times \left(\text{pg}A_{\text{labeled}}^{-}\right)\right]}, \end{array}$$

where *A* ^+^ is the peak area of the (+) enantiomer, *A*_labelled_^+^ is the peak area of the labelled (+) enantiomer, pg*A*_labelled_^+^ is the mass of labeled isomer added in picograms, *A* ^−^ is the peak area of the (−) enantiomer, *A*_labelled_^−^ is the peak area of the labelled (−) enantiomer, and pg*A*_labelled_^−^ is the mass of labelled isomer added in picograms.

*(3) Mass Spectrometric Detection.* Several mass spectrometric techniques were reported for detection of HBCDs. Morris et al. applied both single quadrupole MS and ion trap MS for detection of HBCDs in sediment and biota samples [102]. Although HBCD molecular ion ([M − H]^−^; *m*/*z* = 640.7) was monitored in both techniques, differences in instrumental response to the three studied HBCD diastereomers were observed. *α*-HBCD recorded the highest response using the single quadrupole MS, while the ion trap MS was most sensitive to *γ*-HBCD. Nevertheless, the use of tandem mass (MS/MS) detection in triple quadrupole (QpQ) mass spectrometers provided high sensitivity and very low LODs (≤1 pg on column) for all HBCD diastereomers using the ion transition [M − H]^−^ → Br^−^ [58]. While electrospray ionisation (ESI) source in negative ion mode is the most commonly used interface for HBCD analysis, both atmospheric pressure photoionisation (APPI) [103] and atmospheric pressure chemical ionisation (APCI) [104] sources proved as useful for HBCD detection. Matrix-related ion suppression issues and differences in the response factors to *α*-, *β*-, and *γ*-HBCD diastereomers were identified as the major challenges encountered with MS/MS analysis of HBCDs using ESI. These drawbacks can be overcome by the use of ^13^C- or d_18_-labelled HBCDs (monitored at *m*/*z*652.4 → 79 and 657.6 → 79, resp.) as surrogate and/or recovery standards. The mass labelled standards behave similarly to native HBCDs in the ion source and can compensate for matrix-related effects. Furthermore, both instrumental response and matrix-related ion suppression varied for *α*-, *β*-, and *γ*-HBCDs indicating that a labelled internal standard is required for each isomer to obtain accurate results (Figure 2) [88].

To enhance the sensitivity of LC-ESI-MS/MS analysis of HBCD enantiomers, the formation of Cl^−^ and CH_3_COO^−^ adducts via addition of NH_4_Cl and CH_3_COONH_4_ to the mobile phase was investigated [105]. While both approaches presented a comparable behaviour for the analysis of food samples, the Cl^−^ method (*m*/*z*  676.6 → 640.6) showed higher sensitivity and the LODs (0.2–0.4 pg on column) and LOQs (0.7–1.4 pg on column) were up to 14 times lower than those obtained applying the CH_3_COO^−^ method (*m*/*z*  700.6 → 640.6). Another interesting approach involves the use of Anion attachment atmospheric pressure photoionization (AA-APPI), with 1,4-dibromobutane in toluene as a bromide source for analysis of HBCDs (*m*/*z*  722.6 → 79) in sediment samples. This method offered increased sensitivity and lower limits of detection than APPI. Furthermore, minimal matrix effects were found with AA-APPI in sediment extracts providing a major advantage over ESI-based methods [106].

#### 3.2.2. TBBP-A

Avoiding the derivatisation step of phenolic OH groups required prior to GC/MS analysis of TBBP-A was not the only advantage gained by using LC/MS for determination of this BFR. Another advantage was the possible use of ^13^C-labelled TBBP-A as an internal standard which greatly improves the quality of analytical data obtained via compensation for matrix-related effects that can affect analyte ion intensity [16].

(*1) Stationary Phase.* Several studies have reported the use of C_18_ RP columns with various dimensions and particle sizes for analysis of TBBP-A [16]. In general, TBBP-A is rarely measured alone and is usually included in multiresidue analytical methodology for analysis of various BFRs [107]. Guerra et al. applied a* Symmetry C*_*18*_ *column (2.1 × 150 mm, 5 μm) preceded by a C*_*18*_ *guard column (2.1 × 10 mm) supplied by Waters (Milford, MA, USA)* for baseline separation of TBBPA and related compounds bisphenol A (BPA), monobromobisphenol A (MonoBBPA), dibromobisphenol A (DiBBPA), and tribromobisphenol A (TriBBPA) in sewage sludge and sediment samples [108]. Application of UPLC columns (*Acquity HSS T3, 100 *×* 2.1 mm, 1.8 μm, Waters, MA, USA*) resulted in a shorter retention time (6.5 minutes) than HPLC columns [90].

*(2) Mobile Phase.* Chu et al. reported that, by using methanol as mobile phase, the LC-ESI-MS/MS response factor for TBBP-A was ~one third greater than when acetonitrile was used due to a more stable detector baseline [109]. Similar results were recently reported by Lankova et al. using the Turbo V ion source for UPLC-MS/MS analysis of TBBP-A in fish samples [90]. Therefore, multiresidue analytical methods for determination of TBBP-A with other BFRs applied only methanol/water mobile phase gradients (Figure 2) [90, 93, 108, 110].

*(3) Mass Spectrometric Detection.* Unlike HBCDs, Tollbäck et al. found that ESI source in negative ion mode is the most suitable interface for TBBP-A analysis with LC-MS providing 30–40 times lower LODs than those obtained by APCI [111]. Therefore, LC-ESI-MS/MS in negative ion mode was widely reported for determination of TBBP-A concentrations in various environmental matrices via monitoring the mass transitions corresponding to [M − H]^−^ → Br^−^ (*m*/*z*  542.6 → 79 and 552.6 → 79 for native and ^13^C-TBBP-A, resp.) [16].

Although early studies were focused on the use of triple quadrupole mass spectrometers, the high selectivity of ion-trap MS was applied for the determination of TBBPA in sediment and sewage sludge scanning the range from *m*/*z* 145–543 after LC separation [112]. Guerra et al. described a method based on liquid chromatography/quadrupole linear ion trap mass spectrometry (LC-QqLIT-MS) for separation and quantification of TBBPA and related compounds bisphenol A (BPA), monobromobisphenol A (Mono-BBPA), dibromobisphenol A (Di-BBPA), and tribromobisphenol A (Tri-BBPA) together with *α*-, *β*-, and *γ*-HBCD diastereomers in sewage sludge and sediment samples [108]. The reported method displayed excellent LODs in selective reaction monitoring (SRM) mode (0.1–1.8 pg), but even better results were obtained in enhanced product ion (EPI) mode (0.01–0.5* *pg). Interestingly, desorption atmospheric pressure photoionization-mass spectrometry (DAPPI-MS) in negative ion mode was applied successfully for analysis of TBBP-A in circuit board and orange peel samples using anisole as spraying solvent. This method displayed the advantages of minimal sample treatment and low LOD (0.3 ng/g) [113]. Recently, a different approach was adopted for analysis of TBBP-A in plasma and serum samples using LC-ESI(+)-MS/MS. The method is based on derivatisation of TBBP-A in the extracts using dansyl chloride reagent. The dansylated derivatives are then monitored at *m*/*z*  505.9 → 171.1 and 512.9 → 171.1 for native and ^13^C-TBBP-A with method LOQ as low as 0.03 ng/g [114].

#### 3.2.3. PBDEs and NBFRs

Fewer studies have reported on the analysis of PBDEs and NBFRs using LC/MS techniques. This may be attributed to the presence of well-established, sensitive, and efficient protocols for analysis of these hydrophobic compounds using GC/MS techniques. However, LC/MS analysis can provide a major advantage for analysis of heavy molecular weight BFRs (e.g., BDE-209 and DBDPE) which may undergo thermal degradation and/or extensive fragmentation during the course of GC/MS analysis [58]. LC/MS methods in ESI mode may have limited use for PBDEs due to poor ionization in this source [115]. Abdallah et al. reported an isotope dilution method using ^13^C-labelled internal standards for quantification of 14 major tetra- to deca-PBDEs using LC-NI-APPI/MS/MS. The 14 PBDEs were baseline separated on C_18_-RP column (Pursuit XRS3, 250 × 4.6 mm, 3 *μ*m,* Agilent, CA, USA*) using mobile phase gradient of methanol/toluene and water. The method applied the soft photoionisation technique to obtain stable pseudomolecular ions [M − Br + O]^−^ and [M − 2Br + O]^−^ in Q1 which enabled the use of isotopically labelled internal standard for quantification [116]. The method was then successfully applied for analysis of PBDEs in dust [116], air [117], and human milk samples [118]. Zhou et al. [103] developed a sensitive and high throughput LC–NI-APPI-MS/MS method for the analysis of 36 brominated flame retardants including PBDEs, HBCDs, TBBP-A, and several NBFRs in fish samples. The method used an Ultra-II C_18_ column (100 × 2.1 mm, 2.2 *μ*m, RESTEK, PA, USA) operated at 25°C for separation of target compounds with a methanol/water mobile phase gradient at a flow rate of 400 *μ*L/min. In comparison with acetone, toluene provided around 10% higher ion intensity for less hydrophobic compounds. Three categories of precursor ions were observed in the APPI source: (1) displacement products, for example, [M − Br + O]^−^ and [M − HBr − Br + O_2_]^−^; (2) elimination products, for example, [M − H]^−^ and (3) association product, for example [M + O_2_]^−^. The dominant precursor ion used for quantification of the studied BFRs was [M − Br + O]^−^ [103]. In another study, APCI source was investigated by the same authors for determination of 38 BFRs in wastewater samples. For MS/MS detection, relatively high collision energy was required to produce abundant Br^−^ product ions, and the authors suggested increasing the collision gas pressure may generate more of these ions. The method was simple, sensitive, and applicable to compounds with a wide range of physicochemical properties [119]. Mascolo et al. used a C_18_-BEH column (150 × 2.1 mm, 1.7 *μ*m) for separation of 11 tetra- to deca-PBDEs. The column was kept at 40°C while separation was achieved using a methanol/water gradient. Method LODs as low as 3–198 pg/g and 4–380 pg/g were reported for APCI and APPI (toluene as dopant) sources, respectively. Depending on the PBDE congener, the APCI source was 2–8 times more sensitive than APPI [120].

Letcher and Chu reported the application of LC-NI-APPI-MS/MS for quantification of TBBPA-S-DBPE, TBBPA-AE, and TBBPA-DBPE in herring gull eggs. Target analytes were separated on a ZOBRAX SB-C_18_ column (2.1 × 30 mm, 3.5 *μ*m).The method depends on the use of acetone as both the organic solvent in the mobile phase and the doping agent for the APPI source. The studied compounds were quantified via monitoring *m*/*z*  997.4 → 79, 655.8 → 79, and 975.5 → 79 for TBBPA-S-DBPE, TBBPA-AE, and TBBPA-DBPE, respectively, corresponding to [M + O_2_]^−^ → Br^−^ transition [121]. More recently, TBBPA-AE and TBBPA-DBPE were analysed by APCI-MS/MS after separation on a C_18_ column (150 × 2.1 mm, 5 *μ*m) using methanol/water mobile phase. The studied compounds were monitored at *m*/*z*  582.9 → 526.5 and 742.7 → 526.5 for TBBPA-AE and TBBPA-DBPE, respectively. Method LOD ranged from 10–30 pg/g in various environmental samples [122]. Finally, Arsenault et al. reported an LC-ESI-MS method for analysis of TBECH isomers. TBECH is a novel BFR which has 4 thermolabile diastereomers that can interconvert at temperatures ≥125°C. Incomplete separation of the 4 isomers was performed on a UPLC BEH C_18_ column (2.1 × 100 mm, 1.7 *μ*m) with a methanol/acetonitrile/water gradient. Analytes were detected via monitoring Br^−^ ions in SIM mode due to the lack of molecular ion formation in ESI source [123].

#### 3.2.4. BFR Metabolites

The mounting scientific interest in BFRs in the past few years has resulted in an increasing number of studies on their fate and behaviour in the environment and humans. This lead to the development of analytical methodologies to monitor BFR metabolites and transformation products together with the parent compounds. Since most of the produced metabolites are more polar than the parent BFRs, LC-MS provides a useful, rapid, and sensitive technique for their analyses.

(*1) HBCD Metabolites and Degradation Products.* Abdallah et al. identified 4 isomers of pentabromocyclododecene (PBCD) and two isomers of tetrabromocyclododecadienes (TBCD) as transformation products of HBCDs in indoor dust. These transformation products were separated on a C_18_-RP column (150 × 2.1 mm, 3 *μ*m) using a methanol/water gradient. PBCDs were monitored at 560.6 → 79 while TBCDs were monitored at 480.4 → 79 using an ESI source in negative ion mode. Identification of these transformation products led the authors to hypothesize sequential debromination as a pathway of HBCD transformation [71]. Further studies by the authors using the same analytical method lead to identification of various TBCDs and PBCDs in fish [124] and human milk [110]. HBCD monohydroxylated metabolites were identified by Zegers et al. following in vitro incubation with liver microsomal enzymes of harbour porpoises [125]. The hydroxyl metabolites were separated on a C_18_-RP column (150 × 2.1 mm, 3.5 *μ*m) and monitored at *m*/*z*  656 → 79. Following exposure of female Wistar rats to technical HBCD mixture in feed, Brandsma et al. managed to identify a range of monohydroxyl metabolites of HBCDs, PBCDs, and TBCDs in addition to dihydroxylated PBCD [126]. Tissue extracts were separated into 17 fractions using *μ*Porasil NP-HPLC column (10 *μ*m, 7.8 × 300 mm) prior to analysis by liquid chromatograph with a quadrupole ion trap mass spectrometer (LCQ-MS). The LCQ-MS system featured a Zorbax eclipse XDB-C_18_ column (150 × 2.1 mm, 3.5 *μ*m) preceded by a Zorbax XDB-C_8_ guard column, while a mixture of acetonitrile/0.01 mM ammonium chloride was used as mobile phase. The MS was equipped with an ESI source operated in negative ion mode, while all the target analytes were monitored at *m*/*z* values equivalent to their [M + Cl]^−^ adduct which provided higher sensitivity than the quasimolecular ion species [126]. Hydroxylated metabolites of individual HBCD enantiomers were identified following in vitro incubation with rat liver microsomes [127]. Separation of target analytes was achieved on a combination of Zorbax XDB-C_18_ column (1.8 *μ*m, 150 × 4.6 mm) and a chiral NUCLEODEX *β*-PM (5 *μ*m, 200 × 4.6 mm) analytical column maintained at 15°C using a mixture of methanol/acetonitrile/10 mM ammonium acetate as mobile phase. The mass transitions of 656.7 → 79 and 672.6 → 79 were established to monitor mono- and dihydroxy-HBCD metabolites. More recently, Abdallah et al. optimised a method for simultaneous analysis of HBCDs, PBCDs, TBCDs and their hydroxylated metabolites following in vitro incubation experiments with rat and trout hepatic subcellular (S9) fractions. The method used a combination of a Pursuit XRS3 C_18_ column (150 × 2.1 mm, 3 *μ*m) and a NUCLEODEX *β*-PM (200 × 4.6 mm, 5 *μ*m) chiral column [128]. All target analytes were monitored at MRM corresponding to their respective [M − H]^−^ → Br^−^ mass transitions.

*(2) PBDE Metabolites.* Introduction of one or more hydroxyl groups to PBDEs can result in the formation of more toxic metabolites due to close structural similarity to the thyroid hormones [129]. Hydroxyl PBDE metabolites are nonvolatile, relatively polar compounds which require derivatisation prior to GC/MS analysis. GC-MS methods for analysis of OH-PBDE metabolites must include a derivatization step with diazomethane, which needs to be handled with care due to its explosive characteristics. Furthermore, the efficiency of the derivatization step varies from sample to sample, since the reaction may give a yield less than 100%. Finally, additional sample-preparation or clean-up steps could introduce errors and lengthen analysis time [86]. Therefore, LC-MS is the method of choice for rapid, fast, and sensitive analysis of these compounds [107]. It was reported that ionisation of PBDEs and their metabolites by the ESI source is poor [130]. Therefore, focus has shifted to the application of APCI and APPI sources for their ionisation. Hydroxylated and methoxylated metabolites of tetra-PBDEs were analysed in marine biota using LC-APCI-MS/MS in negative ion mode. Chromatographic separation was performed on a C_18_ analytical column (150 × 4.6 mm, 3 *μ*m) with acetonitrile/water mobile phase. Multiple reaction monitoring (MRM) was performed using the precursor [M − H]^−^ ion for hydroxylated analogs and the [M − Br + O]^−^ ion for tetra-PBDEs and their methoxylated analogs. Method LOQs ranged from 0.11 to 43 ng/g lw [131]. Nine OH-PBDEs, ranging from tri- to hexabrominated were separated and quantified using a similar LC-APCI-MS/MS method. Notably, a significant decrease in ionization was observed in 6-OH-substituted PBDE metabolites with orthosubstituted bromine, relative to the other hydroxylated metabolites. This was attributed to the formation of dioxins as a result of high-temperature conditions in the APCI source, which prevented ionization by hydrogen abstraction. The MS/MS experiments also provided evidence of the neutral losses of HBr and Br2, indicating the possible use of neutral loss scanning and selected reaction monitoring (SRM) for screening of brominated metabolites [86]. Liquid chromatography-electrospray tandem triple quadrupole-linear ion trap mass spectrometer (LC-ESI-QqLIT-MS-MS) in negative mode method was developed for the determination of eleven OH-tri- to OH-hexa-PBDEs [132]. The optimal conditions for proper chromatographic separation of the studied OH-PBDE congeners were the following: Purospher STAR RP-18 endcapped column (125 × 2 mm, 5 *μ*m) working at pH = 10, using ACN and water/methanol 3 : 2 as mobile phase. Selected reaction monitoring (SRM) was used in order to increase sensitivity using transitions corresponding to [M − H]^−^ → Br^−^ for all target metabolites. Instrumental LOQs ranged between 0.6–2 pg on column [132]. APPI was also reported for simultaneous analysis of PBDEs and their hydroxylated metabolites [115]. Following separation on a UPLC Hypersil Gold C_18_ column (100 × 2.1 mm, 1.9 *μ*m) using methanol/water/acetone mobile phase gradient. The optimised method was based on APPI ionization (acetone as dopant) coupled to high-resolution mass spectrometry operating in the full scan mode at a resolution of 60,000 (LTQ-Orbitrap XL mass spectrometer). This provided excellent sensitivity and specificity, allowing the discrimination of signals which could not be resolved on a triple quadrupole used as a reference. The full-scan high-resolution acquisition mode allowed monitoring of both parent PBDEs and their metabolites, including hydroxylated PBDEs, with detection limits ranging from 0.1 to 4.5 pg injected on-column [115]. LC-ESI(+)-MS/MS was reported for analysis of 14 OH-PBDEs in serum following derivatisation with dansyl chloride. Chromatographic separation was achieved on a Luna PFP-2 column (2 × 100 mm, 3 *μ*m) with a mobile phase of water/acetonitrile (both containing 0.1% formic acid). Derivatization and analysis by LC-ESI(+)-MS/MS was reported to produce an intense molecular ion [M + H]^+^ peak and thus a much higher ionization efficiency and yield. Under MS/MS conditions, the dansylated precursor ions also produced an intense fragment ion at *m*/*z* 171 corresponding to the 5-(dimethylamino)-naphthalene moiety. LODs ranged from 0.01 to 014 ng/g for the 14 target OH-PBDEs [114]. An interesting approach involving the use of a comprehensive two-dimensional system coupling UPLC and ion mobility-mass spectrometry (IM-MS) was reported for analysis of 23 mono- to octa-OH-PBDEs. The first-dimensional reversed-phase UPLC was performed on a BEH C_18_ (150 × 2.1 mm, 1.7 *μ*m) chromatographic column using acetonitrile/water gradient elution program with a flow rate ramp. It enabled excellent chromatographic separation for both between-class and within-class OH-PBDEs based on their differences in hydrophobicity. Following the preionization resolution in the first dimension, the second-dimensional IM-MS employed a hybrid electrospray quadrupole ion mobility time-of-flight mass spectrometer and added an extra postionization separation for between-class OH-PBDE congeners on account of their relative mobility disparity during a very short period of 8.8 ms. The two-dimensional separation plane also contributed to the removal of background interference ions and the enhanced confidence in the characterization of OH-PBDEs of interest [133].

## 4. Current Challenges and Future Perspectives

Screening recent literature on methodologies reported for analysis of different BFRs in various environmental matrices has revealed a few challenges highlighted by several authors. While the methods for extraction and clean-up of different BFRs vary slightly according to physicochemical parameters of target analytes, very little is known about these important parameters for NBFRs [14]. Therefore, more validated studies on important physicochemical parameters of NBFRs (e.g., Henry's law constant, water solubility, log⁡⁡*K*_ow_, and air/water partition coefficients) are required to allow for the development of multiresidue analytical methods and to improve the current understanding of the environmental behaviour of these contaminants. This will also be reflected in the sampling strategies adopted to collect these NBFRs from various environmental matrices.

The continuously escalating global interest in monitoring different classes of environmental contaminants implies the need for efficient, rapid, and high throughput analytical methods. The availability of integrated sample-preparation systems (e.g., automated sample extraction with online clean-up and volume reduction systems) makes them an ideal choice to reduce sample-processing time and to achieve the high-throughput analysis required to process large numbers of samples in environmental monitoring programs with a good precision. While current application of such integrated systems in environmental analysis is limited by their high prices that add to the overall cost of analysis, commercial competition and continuous development are likely to expand their applications in the near future.

Considering the rapid advances of MS-based instrumental techniques, development of analytical methods for smaller amounts of sample is desirable. The concept of small sample volume becomes more attractive with the increasing scientific interest in dried blood spot (DBS) analysis for monitoring of various contaminant groups in human blood [134]. Small sample mass is likely to reduce the matrix-related interferences which entail time-consuming clean-up steps. Yet, such approach necessitates high sensitivities and low method LODs which can only be achieved via rigorous optimization of instrumental parameters.

The large number of legacy and novel BFRs in the market together with the limited budget for their analysis in environmental samples necessitates further development of multiresidue analytical methodologies for simultaneous identification/quantification of various classes of BFRs together with other environmental contaminants in the same sample within a reasonable run time via application of advanced hyphenated analytical techniques, for example, GC × GC-TOF/MS, UPLC-MS/MS, UPLC-HRMS.

Finally, the inclusion of new contaminants (e.g., NBFRs) in existing monitoring protocols is recommended. However, this highlights the need for commercially-available reference standards (labelled and unlabelled) for these compounds, together with the certification of appropriate biotic and abiotic reference materials which are necessary to validate the analytical methods developed and produce accurate results.

## Figures and Tables

**Table 1:** Physicochemical parameters of high production volume BFRs.

Chemical name	Acronym	Molecular Formula	Chemical structure	M.Wt (amu)	BP (°C)	Water solubility (*µ*g/L, 25°C)	Vapour pressure (Pa, 25°C)	log⁡*K*_ow_
2,4,4′-TriBDE	BDE 28	C_12_H_7_Br_3_O	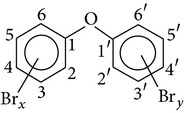	406.90	371	70	1.6 × 10^−5^	5.94
2,2′,4,4′-TetraBDE	BDE 47	C_12_H_6_Br_4_O		485.79	395	11	2.5 × 10^−4^	6.81
2,2′,4,4′,5-PentaBDE	BDE 99	C_12_H_5_Br_5_O		564.69	Decomposes at >300	2.4	5.0 × 10^−5^	6.5–8.4
2,2′,4,4′,6-PentaBDE	BDE 100	C_12_H_5_Br_5_O		564.69	416	40	2.1 × 10^−7^	7.24
2,2′,4,4′,5,5′-HexaBDE	BDE 153	C_12_H_4_Br_6_O		643.58	471	0.9	5.8 × 10^−6^	7.90
2,2′,4,4′5,6′-HexaBDE	BDE 154	C_12_H_4_Br_6_O		643.58	453	1	2.8 × 10^−8^	7.82
2,2′,3,4,4′,5′,6-HeptaBDE	BDE 183	C_12_H_3_Br_7_O		722.48	491	2	3.5 × 10^−9^	8.27
2,2′,3,3′,4,4′,5,5′,6,6′-DecaBDE	BDE 209	C_12_Br_10_O		959.17	Decomposes at >320	<0.1	4.6 × 10^−6^^*^	6.3–12.6
Decabromodiphenyl-ethane	DBDPE	C_14_H_4_Br_10_	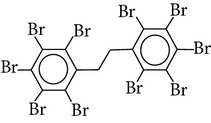	971.22	676	0.72	2.5 × 10^−11^	11.7–13.6
2-Ethylhexyl 2,3,4,5-tetrabromobenzoate	TBB	C_15_H_18_Br_4_O_2_	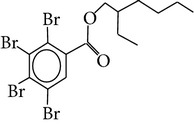	549.92	477	1.1 × 10^−2^	—	7.28–8.75
1,2 bis(2,4,6-tribromophenoxy) ethane	BTBPE	C_14_H_8_Br_6_O_2_	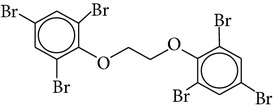	687.64	566	200	3.2 × 10^−8^	8.31–9.15
Bis(2-ethyl-1-hexyl) tetrabromophthalate	TBPH	C_24_H_34_Br_4_O_4_	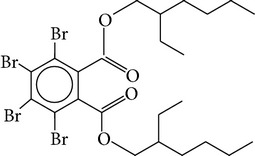	706.14	585	1.2 × 10^−8^	2.3 × 10^−9^	9.34–11.95
Pentabromotoluene	PBT	C_7_H_3_Br_5_	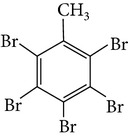	486.62	394	0.9	1.9 × 10^−5^	6.26–6.99
Pentabromoethyl-benzene	PBEB	C_8_H_5_Br_5_	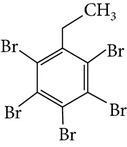	500.68	413	47	6.2 × 10^−4^	7.48
1,2-Dibromo-4-(1,2-dibromoethyl) cyclohexane	TBECH	C_8_H_12_Br_4_	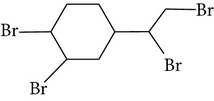	427.84	371	0.07	1.4 × 10^−2^	5.24
Tetrabromobisphenol-A	TBBP-A	C_15_H_12_Br_4_O_2_	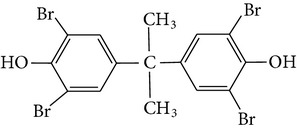	543.9	decomposes at >250	4.16 × 10^−3^	1.76 × 10^−11^	4.50
1,2,5,6,9,10-Hexabrobocyclododecane	HBCD	C_12_H_18_Br_6_	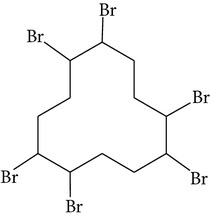	641.7	decomposes at >190	∗∗	6.3 × 10^−5^	5.62

^*^Value reported at 21°C; ^**^isomer specific values: *α*-HBCD: 49; *β*-HBCD: 15; *γ*-HBCD: 2.

**Table 2:** Summary of analytical procedures used for determination of BFRs in various abiotic matrices.

BFRs^*^	Sample matrix	Pretreatment	Extraction	Clean-up	Instrumental analysis	Recovery (%)	Reference
Di- to deca-BDEs, PBT, pTBX, PBEB, HBB, BTBPE, DBDPE, PBBs	Outdoor air	(i) GFF (ii) PUF	(i) Soxhlet (48 hours) (ii) Hexane/acetone (1 : 1)	(i) Silica/alumina column. (ii) Elution with hexane-DCM (1 : 1)	GC-ECNI-MS	67–122 ± 15%	[23]

Di- to deca-BDEs, BTBPE, DBDPE, PBEB, HBB	Outdoor air	(i) Quartz fibre filter (ii) Amberlite XAD-2 resin.	(i) Soxhlet (24 hours) (ii) Hexane/acetone (1 : 1)	(i) 3.5% (w/w) water-deactivated silica gel column. (ii) Elution with 25 mL hexane followed by 25 mL hexane/DCM (1 : 1).	GC-ECNI-MS	61–108 ± 19%	[24]

Tri- to deca-BDEs	Outdoor air	(i) GFF (ii) PUF/XAD-2 resin.	(i) For GFF: ultrasonication with hexane/acetone (1 : 1) for 20 min. (3 times). (ii) For PUF/XAD-2: Soxhlet (24 hours) using hexane/diethyl ether (8 : 2).	(i) Florisil cartridges topped with 0.5 g anhydrous Na_2_SO_4_ eluted with 8 mL hexane. (ii) Multilayer column containing 1 g of each of H_2_SO_4_-impregnated silica (40% w/w), activated silica, and activated neutral alumina. Rinsed with 21 mL hexane (discarded). PBDEs were eluted with 15 mL hexane/DCM (8 : 2).	GC-ECNI-MS	50–85	[25]

Tri- to deca-BDEs, DPTE, HBB, TBPH, PBT, BTBPE, OBIND	Outdoor air	(i) GFF (ii) PUF/XAD-2 resin.	(i) Soxhlet (16 hours) (ii) hexane/DCM (1 : 1)	(i) 10% water deactivated silica column topped with 3 g anhydrous Na_2_SO_4_ (ii) Elution with 15 mL hexane	GC-ECNI-MS	69–73 ± 12%	[26]

Tetra- to deca-BDEs, TBBP-A, *α*-, *β*- and *γ*-HBCDs	Indoor air (cars)	(i) GFF (ii) PUF	(i) Soxhlet (8 hours) (ii) DCM	(i) 8 g H_2_SO_4_-impregnated silica (44% w/w). (ii) Elution with 25 mL hexane/DCM (1 : 1)	LC-APPI-MS/MS	69–107	[27]

Tri- to hepta-BDEs, hydroxyl-BDEs, methoxy-BDEs	Water, soil, sediment		(i) Water: LLE with 25 mL (1 : 1) hexane/MTBE (twice) (ii) Soil/sediment: 20 min ultrasonic extraction with 25 mL (1 : 1) hexane/MTBE followed by 10 min centrifugation (3 times)	(i) H_2_SO_4_-impregnated silica (preceded with 2 g Cu powder for sediment samples). (ii) Elution with 40 mL DCM. (iii) Fractionation on 5 g of 5% deactivated silica topped with 1 g anhydrous Na_2_SO_4._	(i) GC-EI-MS for BDEs and methoxy-BDEs (ii) LC-ESI-MS/MS for hydoxy-BDEs	71–116 ± 14%	[28]

Tri- to deca-BDEs	Water		SFOME using 25 *μ*L 2-dodecanol as organic drop.		HPLC-UV	81–116%	[29]

Mono- to hexa-PBBs	Water		(i) SPE using 100 mg of synthetic MIP loaded on silica gel (ii) Elution with 3.0 mL hexane : DCM (1 : 1)		GC-ECD	70–97	[30]

Tetra- to deca-BDEs	Water, soil, sediment	(i) Water: filtered through 0.45 *μ*m GFF, mixed with 40% methanol and sonicated for 30 min. (ii) Soil/sediment: freeze-dried, sieved (0.2 mm mesh size), grounded, mixed with anhydrous Na_2_SO_4_ and dried.	(i) Water: SPE (C_18 _cartridges). Eluted with 3 mL methanol, 3 mL DCM, and 3 mL hexane. (ii) Soil/sediment: MAE with 20 mL hexane/acetone (1 : 1) at 110°C for 20 min.	(i) Water: packed column with, from bottom to top, 6 cm alumina, 2 cm l silica, 5 cm alkalinized silica, 2 cm silica, 8 cm acidified silica, and 1 cm Na_2_SO_4_. Eluted with 70 mL DCM. (ii) Soil/sediment: ultrasonication for 30 min with Cu powder for sulphur removal followed by sulphuric acid wash and florisil column.	GC-EI-MS	53–130	[31]

Tri- to deca-BDEs, HBCD, BTBPE, PBEB, and DBDPE.	Ice caps	XAD-2 resin columns.	Elution with methanol and DCM.	(i) 10% deactivated silica column. Eluted with 10% methanol in DCM. (ii) Activated silica column. Eluted with hexane then (1 : 1) hexane: DCM.	GC-ECNI-MS	76–93 ± 30%	[32]

Tetra- to deca-BDEs, TBBP-A, *α*-, *β*-, and *γ*-HBCDs	Dust (cars)	Sieving (500 *μ*m mesh size).	PLE using hexane/DCM (1 : 9). Pressure 1500 psi, temp. 90°C, heating time 5 min, static time 4 min, 3 extraction cycles.	(i) 8 g H_2_SO_4_-impregnated silica (44% w/w). (ii) Elution with 25 mL hexane/DCM (1 : 1)	LC-APPI-MS/MS	71–105	[33]

Tri- to deca-BDEs, *α*-, *β*-, and *γ*-HBCDs, BTBPE, DBDPE, HCDBCO, TBB, TBPH.	Dust (house)	Sieving (500 *μ*m mesh size).	(i) Vortexing with 2 mL hexane/acetone (3 : 1) for 2 min. (ii) Ultrasonic extraction for 5 min. followed by centrifugation at 3500 rpm for 2 min (3 times).	(i) Florisil cartridge. Elution with 8 mL hexane (fraction1; BDEs, HBCDs, BTBPE, DBDPE, HCDBCO, TBB) then 10 mL EtAc (TBPH and HBCDs). (ii) Fraction 1 is further cleaned up on H_2_SO_4_-impregnated silica (44% w/w). Elution with 10 mL hexane/DCM (1 : 1)	(i) GC-ECNI-MS (ii) LC-ESI-MS/MS for HBCDs only.	69–122	[34]

Tri- to deca-BDEs	Dust (SRM)	Dust wet with DCM (2 : 3 w/w)	SFE using 1,1,1,2-tetrafluoroethane. 204 atm and 200°C.		LC-APPI-MS/MS	86 ± 6%	[35]

Di- to deca-BDEs, *α*-, *β*-, and *γ*-HBCDs, BTBPE, DBDPE	Soil		(i) Shaking for 60 min with acetone/hexane (1 : 1) (ii) Ultrasonication for 15 min (iii) Centrifugation (2500 rpm, 10 min)	(i) Multilayer column (silica gel, 2% KOH-impregnated silica, 44% and 22% H_2_SO_4_-impregnated silica, Na_2_SO_4_). Eluted with hexane/DCM (3 : 1) (ii) GPC	(i) GC-ECNI-MS (ii) LC-ESI-MS/MS for HBCDs only.	26–119	[36]

Tri- to deca-BDEs	Soil	(i) Sieving (2 mm mesh size) (ii) Dispersion in anhydrous Na_2_SO_4_ (iii) Air drying.	QuEChERS: vortexing for 2 min with 25 mL DCM followed by ultrasonication for 20 min the centrifugation at 5000 rpm for 5 min (2 times)	Alumina SPE cartridges. Elution with 6 mL DCM.	GC-ECNI-MS	61–107	[37]

Tetra- to hexa-BDEs	Sediment	(i) Freeze drying (ii) Sieving (0.3 mm wire mesh)	(i) SPME using polyacrylate coated fibre in the headspace mode at 100°C for 40 min. (ii) Simultaneous oxidation with KMnO_4_ and H_2_SO_4_. (iii) Thermal desorption of fibre at 300°C for 2 min.		GC-MS/MS	76–111	[38]

TBB, TBECH, TBCT, PBT, HBB, PBEB, PBBA, PBBB, HCDBCO, PBB153, *α*-, *β*-, *γ*-HBCDs, BTBPE, DBDPE	Sediment	(i) Air drying. (ii) Dispersion in anhydrous Na_2_SO_4_	(i) Soxhlet (24 hours) (ii) Hexane/DCM (1 : 1)	(i) Cu powder to remove sulphur. (ii) Multilayer column (activated silica, 40% H_2_SO_4_-impregnated silica, activated silica, anhydrous Na_2_SO_4_) (iii) Elution with hexane/DCM (5 : 1)	GC-ECNI-MS	79–87 ± 12 %	[39]

Tri- to deca-BDEs, HBB, BTBPE, TBB, TBPH	(i) Sludge (ii) Biosolids	Dispersion in anhydrous Na_2_SO_4_	PLE using hexane/DCM (1 : 1). Pressure 1500 psi, temp. 100°C (3 cycles)	(i) GPC (ii) Sulphuric acid wash.	(i) GC-EI-MS (HBB and BDE-209) (ii) GC-ECNI-MS.	79 ± 12%	[40]

Tri- to deca-BDEs, TBECH, PBT, HBB, PBEB, HCDBCO, TBB, TBPH, BTBPE, DBDPE	Sediment, sludge, dust		UAE for 10 min with ethylacetate/cyclohexane (5 : 2).	Florisil columns and activated copper to remove sulphur from sediment samples.	GC-MS/MS	69–140	[41]

^*^Decabromodiphenyl ethane (DBDPE) and 1,2-bis(2,4,6-tribromophenoxy)-ethane (BTBPE), pentabromoethylbenzene (PBEB), 2,3,5,6 tetrabromo-p-xylene (pTBX), pentabromotoluene (PBT), hexabromobenzene (HBB), polybrominated biphenyls (PBBs), 2,3-dibromopropyl-2,4,6-tribromophenyl ether (DPTE), octabromotrimethylphenylindane (OBIND), bis(2-ethylhexyl)-tetrabromophthalate (TBPH), 2-ethylhexyl-2,3,4,5-tetrabromobenzoate (TBB), hexachlorocyclopentadienyldibromocyclooctane (HCDBCO), tetrabromo-o-chlorotoluene (TBCT), pentabromobenzyl acrylate (PBBA), allyl 2,4,6-tribromophenylether (ATE), 1,2-dibromo-4-(1,2-dibromoethyl)cyclohexane (TBECH), pentabromobenzyl bromide (PBBB).

**Table 3:** Summary of analytical procedures used for determination of BFRs in various biotic matrices.

BFRs^*^	Sample matrix	Pretreatment	Extraction	Clean-up	Instrumental analysis	Recovery (%)	Reference
PBBs (15, 31, 49, 52, 77, 101, 103, 153, 155, 169)	Fish tissue (trout, salmon, horse mackerel, sardine, and gilthead sea bream)	(i) Freeze drying (ii) Equilibration overnight (iii) Na_2_SO_4_ addition and mixing (3 : 1, w/w, ratio with sample) (iv) Addition of 15 g of acidified silica (44%, w/w)	(i) PLE at 100°C with *n*-hexane (ii) 3 static cycles of 5 min each and a flush volume of 60%	In-cell clean-up (see pretreatment)	GC-IT-MS/MS	50–95%	[42]

PBDEs (28, 47, 66, 85, 99, 100, 153, 154, 183), MeO-PBDEs	Fish and shellfish	(i) Sample lyophilisation (ii) Equilibration overnight (iii) Addition of 25 g of activated Florisil (fat retainer) and 10 g of anhydrous Na_2_SO_4_	(i) PLE at 100°C with DCM/*n*-hexane (1/9, v/v), 1500 psi (ii) 3 static cycles of 5 min each and a flush volume of 100%	In-cell clean-up (see pretreatment)	GC-IT-MS/MS	88–98% 90–98%	[43, 44]

HBCDs	Edible seaweed	Sample grinding	(i) PLE at 80°C with EtAc at 1500 psi (ii) 2 static cycles of 3 min each and a flush volume of 60%	(i) Columns with neutral alumina (3% deactivated), neutral silica (3% deactivated), and Na_2_SO_4_ (ii) Elution with 25 mL *n*-hexane	LC-ESI-MS/MS	93–103%	[45]

PBDEs (47, 99, 100, 153)	Fish	Sample grinding with anhydrous Na_2_SO_4_	US extracted for 30 min with 8 mL of DCM/*n*-hexane (1/4, v/v)	Extract mixed with C_18_-silica, vortexed, and centrifuged	GC-EI-MS/MS	75–114%	[46]

PBDEs (47, 99, 100, 85, 154, 153)	Plastic bottled beverages	Degassed for 10 min in an ultrasonic bath at ambient temperature	Dispersive solid-phase extraction with 4 mL acetonitrile, with 6 g of anh MgSO_4_ and 1.5 g of NaCl	Dispersive liquid-liquid microextraction with 50 mg primary amine silica (PSA) for green tea beverage, 50 mg C_18_ for carbonated beverage, and a mixture of 50 mg PSA and 25 mg C_18_ for orange juice	GC-EI-MS	85–115%	[47]

PBDEs (47, 85, 99, 100, 153, 154)	Milk (supermarket and raw bovine milk)	(i) 5 mL of 50% (w/v) NaOH and 1 mL of acetone (ii) The mixture was further heated at 70°C in water bath	Dispersive liquid-liquid microextraction with 5 mL *n*-hexane	LC-Florisil column: elution with *n*-hexane, solvent exchanged to acetone and water and further extracted in chlorobenzene	GC-EI-MS	73–98%	[48]

PBDEs, HBCDs, PBT, HBB, PBEB, BTBPE, DBDPE	Chicken eggs	Lyophiliztion	Soxhlet extraction with hexane/acetone (1 : 1) for 48 h	(i) Gel permeation chromatography (ii) Multilayer silica gel column packed with neutral silica and acidified silica.	GC-ECNI-MS	84–138%	[49]

17 PBDEs and 30 NBFRs	Blubber of harbour porpoises		PLE with hexane/acetone (1 : 1) at 100°C and 120 bar.	(i) Gel permeation chromatography (ii) Florisil column	GC-MS/MS	70–120%	[50]

PBDEs (3, 15, 28, 47, 77, 99, 100, 118, 126, 153, 183)	Venous and umbilical cord blood sera (human)	Addition of 3 mL of concentrated H_2_SO_4_	Multiple liquid-liquid extraction with *n*-hexane (total of 7 mL solvent)	Addition of 2 mL of concentrated H_2_SO_4_	GC-ECNI-MS	90–120%	[51]

PBDEs (47, 99, 100, 153, 183)	(i) Human blood (50 *µ*L) (ii) Dried blood spot (human)	(i) 2 h for equilibration, formic acid : acetone (3/2, v/v) for protein denaturation. (ii) Cut into small pieces, formic acid : acetone (2/3, v/v) for protein denaturation.	(i) LLE with DCM/*n*-hexane (1/4, v/v) and repeated with *n*-hexane followed by combination of organic solvents. (ii) ultrasonication with DCM/*n*-hexane (4/1, v/v) and repeated with *n*-hexane followed by combination of organic solvents.		GC-EI-HRMS	(i) 71–99 (ii) 75–120%	[52]

TBBP-A, *α*-, *β*-, and *γ*-HBCDs	Human milk	Freeze-drying	PLE using hexane/DCM (1 : 9). Pressure 1500 psi, temp. 90°C, heating time 5 min, static time 4 min, 3 extraction cycles.	(i) Wash with concentrated sulphuric acid. (ii) Florisil/Na_2_SO_4_ column.	LC-MS/MS	78–109%	[43]

PBDEs (47, 85, 99, 100, 153, 154, 183, 196, 197, 203, 206, 207, 208, 209)	Human milk	Freeze-drying	PLE using hexane/DCM (1 : 9). Pressure 1500 psi, temp. 90°C, heating time 5 min, static time 4 min, 3 extraction cycles.	(i) Wash with concentrated sulphuric acid. (ii) Florisil/Na_2_SO_4_ column.	LC-MS/MS	74–112%	[53]

^*^For details on compounds' abbreviation, see Table 2.
